# Alternative transcripts of the SERPINA1 gene in alpha-1 antitrypsin deficiency

**DOI:** 10.1186/s12967-015-0585-y

**Published:** 2015-07-04

**Authors:** Nerea Matamala, Maria Teresa Martínez, Beatriz Lara, Laura Pérez, Irene Vázquez, Azucena Jimenez, Miguel Barquín, Ilaria Ferrarotti, Ignacio Blanco, Sabina Janciauskiene, Beatriz Martinez-Delgado

**Affiliations:** Molecular Genetics Unit, Instituto de Investigación de Enfermedades Raras (IIER), Instituto de Salud Carlos III (ISCIII), Carretera Majadahonda-Pozuelo Km 2,200, 28220 Majadahonda, Madrid Spain; Pneumology Department, Hospital 12 de Octubre, Madrid, Spain; Respiratory Medicine Department, Royal Exeter and Devon Hospital, Exeter, Devon UK; Section of Pneumology, Department of Molecular Medicine, Center for Diagnosis of Inherited Alpha-1 Antitrypsin Deficiency, IRCCS San Matteo Hospital Foundation, University of Pavia, Pavia, Italy; Alpha1-Antitrypsin Deficiency Spanish Registry, Lung Foundation RESPIRA, Spanish Society of Pneumology (SEPAR), Barcelona, Spain; Department of Respiratory Medicine, Hannover Medical School, Hanover, Germany; Biomedical Research in Endstage and Obstructive Lung Disease Hannover (BREATH), Member of the German Center for Lung Research (DZL), 30626 Hanover, Germany

**Keywords:** Alpha-1 antitrypsin, Alpha-1 antitrypsin deficiency, SERPINA1, Transcription, Alternative splicing, Quantitative PCR

## Abstract

**Background:**

SERPINA1 is the gene for alpha-1 antitrypsin (AAT), an acute phase protein with anti-protease and immunoregulatory activities. Mutations in SERPINA1 gene cause AAT deficiency and predispose individuals to early-onset emphysema and liver diseases. Expression of the SERPINA1 gene is regulated by different promoters and alternative splicing events among non-coding exons 1A, 1B and 1C.

**Methods:**

We have developed three quantitative PCR (QT-PCR) assays (1A, 1B and 1C). These assays were applied for the analysis of SERPINA1 alternative transcripts in: (1) 16 human tissues and (2) peripheral blood leukocytes from 33 subjects with AAT mutations and 7 controls.

**Results:**

Tissue-specific expression was found for the SERPINA1 transcripts. The 1A transcripts were mainly expressed in leukocytes and lung tissue while those detected with the 1B assay were highly restricted to leukocytes. Only 1B transcripts significantly correlated with serum AAT levels. The 1C transcripts were specifically found in lung, liver, kidney and pancreas. Furthermore, the expression of transcripts was related to AAT genotypes. While deficient variants of AAT had no pronounced effect on the transcript expression, null alleles were associated with significant reduction of different transcripts.

**Conclusions:**

The possibility to discriminate between SERPINA1 alternative splicing products will help us to understand better the regulation of SERPINA1 gene and its association with SERPINA1 mutations-related diseases.

**Electronic supplementary material:**

The online version of this article (doi:10.1186/s12967-015-0585-y) contains supplementary material, which is available to authorized users.

## Background

Human alpha-1 antitrypsin (AAT), also named alpha-1 proteinase inhibitor (α1-Pi) is the prototypic member of the serine protease inhibitor (SERPIN) superfamily of proteins [1]. AAT is codified by the SERPINA1 gene, which is located on human chromosome 14q32 [2–4], mapped in the same cluster together with other serpin genes, SERPINA3 (α1-antichymotrypsin), SERPINA4 (kallistatin precursor), SERPINA5 (PCI, protein C inhibitor), SERPINA9 (centerin), SERPINA10 (ZPI, protein Z-dependent protease inhibitor) as well as SERPINA11 [5]. SERPINA genes have similarities in their structure and characteristically consist of four exons with identical positioning and phasing of the intron–exon boundaries. Interestingly, in spite of their close proximity, the corresponding proteins have different functions, raising important questions about the evolution of gene function and expression.

The structure of SERPINA1 is organized into three non-coding exons (1A, 1B and 1C), four coding exons (2–5) and six introns [2–4]. Expression of the SERPINA1 gene is controlled predominantly by two promoters in the 5′ untranslated region (5′UTR) regulatory region. The hepatocyte SERPINA1 promoter is located upstream of the hepatocyte transcription start site, within exon 1C [4, 6, 7]. An alternative promoter region, located upstream of exon 1A, controls SERPINA1 expression in other cells, such as monocytes and macrophages [7]. Therefore, the primary transcript generated in hepatocytes is smaller than two other major transcripts generated from exons 1A and 1B. At least two different transcription start sites are located within the exon 1A, typically used in monocytes, whereas a third transcription site is located before exon 1B [8–10]. Additional transcription initiation sites for SERPINA1 gene expression were proposed for corneal cells and A549 lung cell line [11–13].

The transfer of the genomic information from DNA to RNA to protein is a highly complex process due to the generation of alternative gene products from a single gene locus [14]. This latter can occur through transcriptional or post-transcriptional (splicing) mechanisms [15]. Thus, almost all multi-exon human genes can generate multiple mRNA/transcript variants, which may encode isoforms having different biological functions [16, 17]. Studies on alternatively spliced isoforms suggested that changes in splicing are related to gene function and specific diseases [18, 19]. Although splicing is an abundant phenomenon [20], characterization of splicing isoforms has not been performed for many genes, including SERPINA1 gene.

In the SERPINA1 gene, different transcripts are produced due to the use of different transcription initiation sites, and within non-coding exons (1A, 1B and 1C) the alternative splicing events occurring in a stimulus- and cell-type specific manner [7, 8, 21]. These facts highlight the importance of the 5′UTR region for the regulation of AAT expression.

AAT is expressed mainly by hepatocytes [9] although other cell types, including monocytes, macrophages, intestinal epithelial cells or cornea, also express this protein [12, 22–25]. The level of plasma AAT is controlled by a pair of co-dominant alleles and more than 100 variants have been described in the SERPINA1 gene [26]. For clinical purposes AAT variants are classified into four major categories: normal, deficiency, null and dysfunctional. The most common allele is M resulting in a normal concentration of functionally active protein. Reduced levels of the AAT protein are commonly associated with deficient S and Z alleles [27]. There are also other rare deficient variants of the gene and cases with null alleles where no protein is produced due to the nonsense, frameshift or splicing mutations leading to truncated proteins, unstable transcription or mRNA degradation. SERPINA1 gene produces several transcription products of putative clinical relevance. Therefore, it is of great interest to detect and quantify these products in different tissues, and to explore the effect of genetic variants of SERPINA1 on transcription levels of the gene. Quantitative real-time reverse transcription-PCR was used before to study SERPINA1 expression under different experimental conditions and to assess the effect of gene mutations on transcription [28, 29]. However, the quantification of the different SERPINA1 splicing isoforms has not been performed to date. Therefore, the development of methods to quantify the abundance of these isoforms, and the characterization of the splicing pattern in different tissues remains of great importance.

Quantitative real-time PCR (QT-PCR) is a “gold” standard for mRNA quantification and has occasionally been used for quantification of specific splicing isoforms [19, 30–33]. The aim of this study was to develop a QT-PCR approach for measuring the expression level of the alternative transcripts of the SERPINA1 gene. Three different QT-PCR assays directed to non-coding exons 1A, 1B and 1C were designed allowing quantification of specific transcripts. In order to evaluate the clinical relevance of alternative splicing isoforms, the expression of these transcripts was assessed in a set of different human tissue samples and in the peripheral blood samples from AAT deficiency subjects with different genetic mutations.

## Methods

### Material from human tissues

Commercially available cDNA from human tissues (Human MTC Panel I and II, Clontech) were used to measure the expression of SERPINA1 transcripts in a total of 16 different human tissues including heart, brain, placenta, lung, liver, skeletal muscle, kidney, pancreas, colon, ovary, peripheral leukocytes, prostate, small intestine, spleen, thymus. Two or more different experiments were performed in triplicate for all tissues. Expression in tissues was normalized to the expression obtained in a peripheral leukocyte sample.

### AAT deficiency subjects samples

Peripheral blood samples were obtained from 33 AATD subjects evaluated in the Spanish Registry of Patients with AAT Deficiency or in the Center for Diagnosis of Inherited Alpha-1 Antitrypsin Deficiency in Pavia, Italy, and seven healthy individuals with normal (MM) AAT genotype that were used as controls. All cases had previously been genotyped by sequencing of all coding exons to characterize the mutation present in each case. Signed informed consent for the study was obtained from all the subjects and the study was approved by the ethics committee of Instituto de Salud Carlos III. Cases with deficient genotypes were members of a total of 18 families carrying the common deficiency variants (S and Z), the rare deficiency variants MMalton and MProcida or the null alleles QOMattawa, QOPorto, QOMadrid, QOBrescia or MVarallo, in heterozygous or homozygous state (Table 1). Among carriers of mutations, 20 presented with lung disease and 13 were asymptomatic blood relatives identified by family screening.

**Table 1:** Genotype and AAT serum levels of the cases analyzed

	ID	Genotype	AAT serum (mg/dl)	Reason for diagnosis
1	14S20	MM	133	Healthy control
2	12OCT02	MM	185	Healthy control
3	12OCT03	MM	124	Healthy control
4	12OCT05	MM	120	Healthy control
5	12OCT07	MM	132	Healthy control
6	12OCT08	MM	170	Healthy control
7	GM1	MM	102	Healthy control
8	12S01	MZ	70	Lung disease
9	12OCT01	ZZ	18	Lung disease
10	12OCT04	ZZ	23.6	Lung disease
11	12OCT06	ZZ	38	Lung disease
12	12OCT09	ZZ	11.5	Lung disease
13	14S19	ZZ	20	Lung disease
14	14S31	ZZ	16.1	Lung disease
15	13S09	M/MMalton	63.2	Lung disease
16	13S12	M/MMalton	75.9	Lung disease
17	13S13	M/MMalton	84.7	Family screening
18	14S27	M/MMalton	70	Family screening
19	14S28	M/MMalton	74	Family screening
20	14S30	M/MMalton	76	Family screening
21	14S29	MMalton/MMalton	6	Lung disease
22	13S03	S/MMalton	67.2	Lung disease
23	13S14	Z/MMalton	45	Lung disease
24	14S23	MProcida/MProcida	17	Lung disease
25	14S25	S/MProcida	49	Family screening
26	14S34	S/MProcida	70	Family screening
27	14S24	M/MProcida	69	Lung disease
28	14S35	M/MProcida	83	Family screening
29	14S36	M/MProcida	81	Family screening
30	14S37	M/MProcida	70	Family Screening
31	13S05	M/QOPorto	72	Family screening
32	13S06	M/QOPorto	67	Family screening
33	13S17	M/QOPorto	65	Family screening
34	13S07	QOPorto/QOMadrid	8.7	Family screening
35	13S08	QOPorto/QOMadrid	9	Lung disease
36	13S16	QOPorto/MProcida	33.4	Lung disease
37	13S02	M/QOMattawa	73.7	Lung disease
38	15S42	QOBrescia/QOBrescia	<10	Lung disease
39	15S43	QOBrescia/QOBrescia	<10	Lung disease
40	15S44	Z/Mvarallo	<10	Lung disease

AAT serum levels were previously determined by nephelometry. Genotypes and serum AAT levels are described in Table 1.

### RT-PCR to analyze alternatively spliced products

Expression of different transcripts in tissues and peripheral blood white cells was evaluated by conventional reverse transcription polymerase chain reaction amplification (RT-PCR). Total RNA extraction was performed using RNAeasy kit (Qiagen, Hilden, Germany). Afterwards, cDNA synthesis was carried out by reverse transcription using the Maxima First Strand cDNA Synthesis kit (Thermo Scientific, Fermentas Life Sciences, St. Leon-Rot, Germany). Since alternative splicing can occur within non-coding exons (1A, 1B and 1C) generating different transcription products, in order to amplify the region between 1A-E2, 1B-E2 and 1C-E2, we used previously designed forward primers located in exons 1A, 1B, and 1C as well as a reverse primer in exon 2 [28]. PCR was performed on 1 μl of cDNA template and 100 ng of each primer. The amplification conditions were as follows: 35 cycles of denaturation at 94°C for 45 s, annealing at 60°C for 30 s, and extension at 72°C for 30 s. PCR products were separated by electrophoresis and visualized on gel-red stained 2% agarose gels.

### Quantitative PCR (QT-PCR) of SERPINA1 transcripts

**Figure 1:**
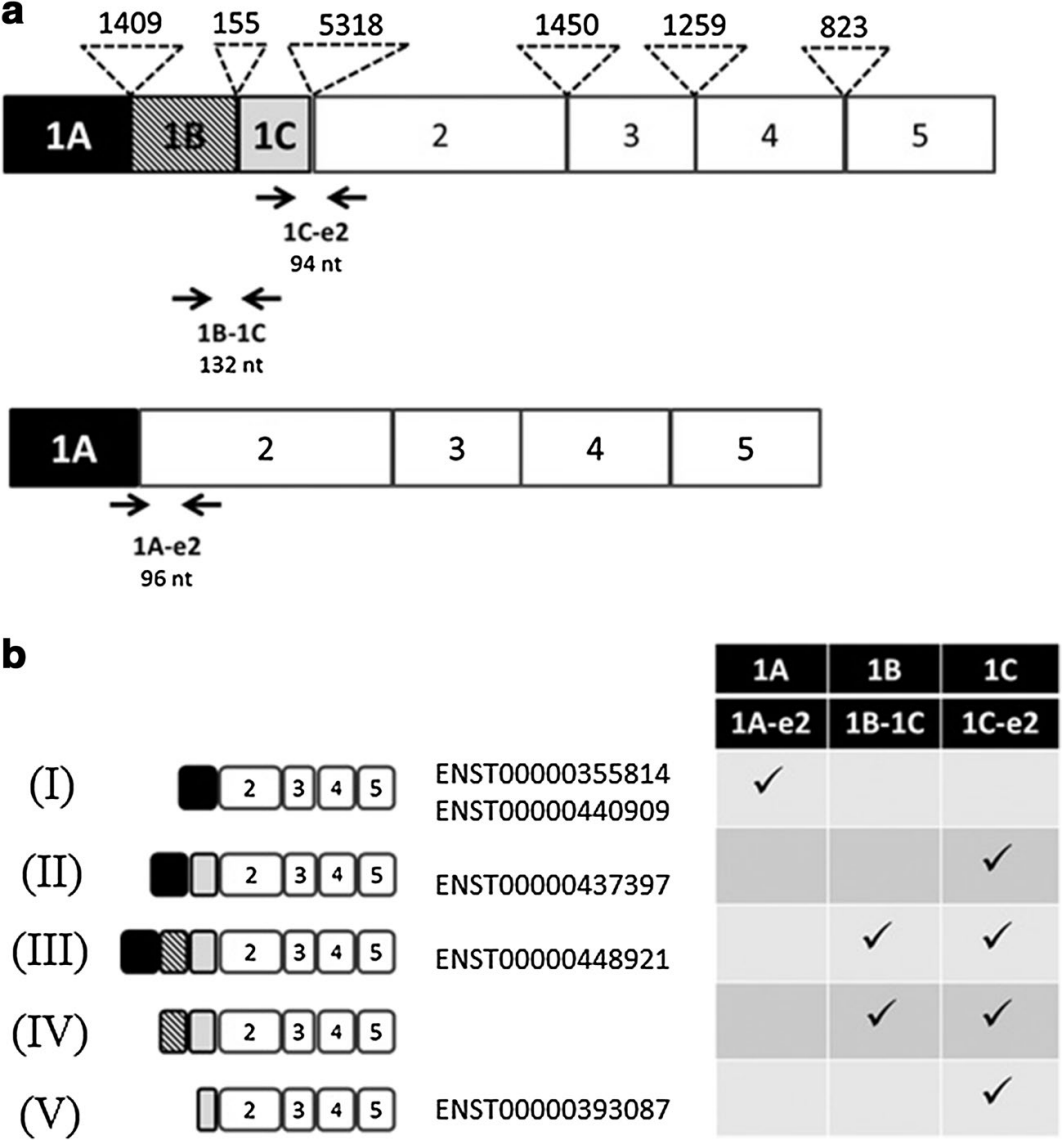
Representation of SERPINA1 gene and QT-PCR assays. **a** Schematic representation of the SERPINA1 gene exons with non-coding exons (1A, 1B and 1C) and exons 2–5 coding for the AAT protein. *Numbers* above the non-coding and coding exons represent the intron length in bp. Locations of the different primers used for the three QT-PCR assays were represented by *arrows*. **b** Different alternative transcripts occur in the AAT gene by alternatively splicing of the non-coding exons 1A, 1B and 1C and the use of the different transcription start sites. Five transcripts (I, II, III, IV and V) can be detected with any of the assays (1A, 1B and 1C). The exon structure of these transcripts corresponds to isoforms found in Ensembl database listed in the Figure. Additionally, another transcript structure (IV), generated by transcription from exon 1B, is not represented in Ensembl database but transcription from exon 1B have been described in the literature [8–10].

We quantitatively analyzed the expression of transcripts containing exon 1A, 1B or 1C. Three different assays (1A, 1B and 1C) were defined (Figure 1; Additional file 1). For each assay specific primers were designed to amplify regions 1A-E2, 1C-E2 and 1B-1C, and specific fluorescent labelled Taqman probes were selected (Universal probe library, UPL, Roche) (Figure 1a). To specifically amplify transcripts with the 1A-E2 junction, a boundary-spanning primer for the sequence encompassing the exon 1A to exon 2 junction (1Ae2_F:5′TGAGGAGAGCAGGAAAGGACA3′) and a reverse primer in exon 2 (1Ae2_R:5′CTCAGCCAGGGAGACAGG3′) together with the probe #18, were used. Thus, with the 1A assay we detected expression of transcript I (Figure 1b).

Assay 1C was designed to amplify a region between exon 1C and exon 2. Junction between exon 1C to E2 is present in different transcription products and therefore it was not possible to design primers able to distinguish between splicing isoforms containing this region (Figure 1b). For this assay primers 1Ce2F: 5′ TTAAATACGGACGAGGACAGG 3′ and 1Ce2R: 5′ACGAGACAGAAGACGGCATT3′, and the probe #73 were used. Another quantitative assay was designed to measure the expression of transcripts specifically containing exon 1B and 1C junction, using a forward primer in 1B (1B1C_F:5′CAGCTAAGTGGTACTCTCCCAGA3′) and a reverse primer in 1C exon (1B1C_R:5′TCGTCCGTATTTAAGCAGTGG3′) in combination with the probe #39.

The cDNA samples from patients and normal cases were diluted and amplified using Taqman Fast advance master mix in a 7500 Fast Real Time PCR System (Applied Biosystems) with recommended PCR cycle conditions. Two genes (ACTB and GUSB) were used as endogenous controls to normalize the expression levels of SERPINA1 transcripts. All experiments were performed in triplicate and data were managed using the Applied Biosystems 7500 software v2.0. Relative expression was calculated by using the comparative Ct method and obtaining the fold-change value (ΔΔCt). The ΔCt value of each sample was calculated using ACTB and GUSB, as an endogenous control genes. An inter-plate calibration sample was used to compare samples analyzed in different PCR plates.

### Statistical analysis

Kolmogorov–Smirnov test was used to analyze the normal distribution of the data and bilateral t test was applied using SPSS version 22 (SPSS Inc, Chicago, IL, USA). Nominal two-sided p values <0.05 were considered statistically significant. Correlation between expression levels of 1A, 1B and 1C and serum levels of AAT were estimated by Pearson’s coefficient. P values below 0.05 were considered as statistically significant.

## Results

### QT-PCR assays for detection of alternative spliced SERPINA1 isoforms

To quantify specific splicing transcripts of the SERPINA1 gene we developed a novel QT-PCR method (Figure 1). For this purpose exon–exon boundary spanning primer and primers located in alternative spliced exons 1B and 1C were designed. Figure 1b shows the different splicing transcripts that are detected using each of the QT-PCR assays (1A, 1B and 1C).

Only transcript I contains the exon 1A directly joined to exon 2. Therefore, we employed a simple strategy using a primer spanning the exon 1A to exon 2 junction, and as illustrated in Figure 1b, the 1A assay specifically quantifies the expression of transcript I. On the other hand, the boundary between exon 1C and exon 2 is not specific and it is included in several transcript variants (II, III, IV and V), which makes impossible individual quantification of transcripts containing this region. Therefore, the 1C assay amplifies and simultaneously detects several transcripts II, III, IV and V. Similarly, the 1B assay, which is developed to detect and quantify the boundary between exon 1B and exon 1C, allows only the combined quantification of transcripts III and IV (Figure 1b).

### Pattern of expression of SERPINA1 isoforms in human tissues

**Figure 2:**
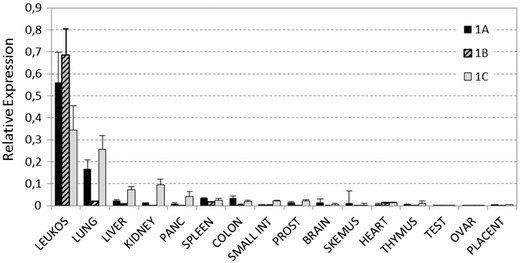
Transcription analyses of SERPINA1 transcripts in human tissues. Relative expression levels of 1A, 1B and 1C transcripts in 16 human tissues is shown. Specific patterns of transcript expression were detected in different tissues. Leukocytes exhibited higher expression levels of transcripts 1A, 1B, and 1C when compared to other tissues. The 1A transcript was found predominantly in leukocytes and lung tissue. Specific expression of 1C transcripts were detected in lung, liver, kidney or pancreas, suggesting that transcription in these tissues start in the transcription site located in exon 1C. Expression of 1B transcripts were almost exclusively detected in leukocytes.

The expression levels of different SERPINA1 transcripts were measured in a panel of various human tissues. Compared to other tissues, leukocytes showed a highest expression of the 1A, 1B and 1C transcripts (Figure 2). The transcript I, specifically detected with the 1A assay, was mainly expressed in leukocytes and lung tissue but it was also detected in liver, colon, spleen, prostate, kidney or brain at much lower level (Figure 2). In addition to leukocytes, 1C transcripts were also expressed in lung, liver and kidney and less abundantly expressed in pancreas and small intestine (Figure 2). The expression of transcripts detected by using 1B assay was almost exclusively restricted to leukocytes, although low expression was also found in lung and spleen. The commercial cDNA samples represent a pooled preparation from many individuals, therefore it is not possible to know which specific cell in the tissue samples is expressing the AAT transcripts. Further experiments using isolated cells remain to be performed.

### SERPINA1 expression in leukocytes from peripheral blood by RT-PCR

**Figure 3:**
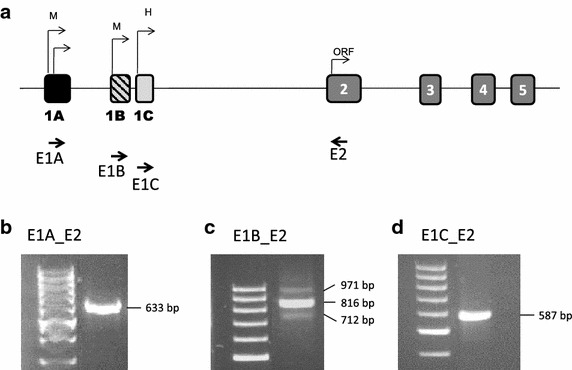
Representative expression of alternative transcripts in blood samples detected by RT-PCR. **a** Schematic graphic of the structure of the SERPINA1 gene. Primers used for RT-PCR expression analysis are represented by *arrows*. RT-PCR expression analysis in leukocytes from peripheral blood samples showed evidence for different alternative splicing event occurring within noncoding exons 1A, 1B and 1C of SERPINA1 gene. **b** Amplification fragment corresponding to the expression in blood samples detected using a forward primer in exon 1A and a reverse primer in exon 2. A single band of 633 bp was detected which corresponded to a transcript containing exon 1A joined directly to exon 2. **c** Bands detected by using forward primer 1B. Different expression fragments were detected corresponding to splicing variants. The structure of these splicing variants has been described in detail before [28]. Three different bands are clearly visualized due to the different splicing sites used within exons 1B and 1C. The band of 712 bp corresponded to the exon 1B joined to exon 2. The band of 816 bp corresponded to an isoform containing both exons 1B and 1C. The upper band of 971 corresponds to a fragment with retained intron in between exons 1B and 1C. **d** Using a forward primer in the exon 1C a single band of 587 bp was amplified.

White blood cells isolated from peripheral blood samples were used to analyze SERPINA1 expression. Since alternative splicing occurs between exons 1A, 1B and 1C, we used forward primers located in these exons and antisense primers in exon 2 (Figure 3). We previously found a unique expression fragment [28] corresponding to a transcript, in which the exon 1A is directly spliced to join exon 2 by skipping of the exons 1B and 1C (Figure 3b). This transcript was initiated in any of the two transcription sites described within exon 1A [7]. However, with the primer located in exon 1B, several transcription products of different size were observed, reflecting the use of the different splicing sites located within exons 1B and 1C (Figure 3c). These products most likely begun in the initiation site described within exon 1B [7] since no alternative transcripts including exon 1B or 1C were detected using the primer in exon 1A. Finally, as expected, amplification using primer in exon 1C generated only one fragment corresponding to joined exon 1C to exon 2 (Figure 3d). This region is present in all transcripts containing the exon 1C. Hence, we cannot determine whether its expression derived from the transcription site within exon 1C or within exons 1A and 1B. Since our analysis was performed on white blood cells, this region most likely corresponds to transcripts starting at any of the monocyte transcription sites. Therefore, we think that leukocytes have active 1A and 1B transcription start sites.

### Effect of SERPINA1 mutations on alternative transcripts expression

**Figure 4:**
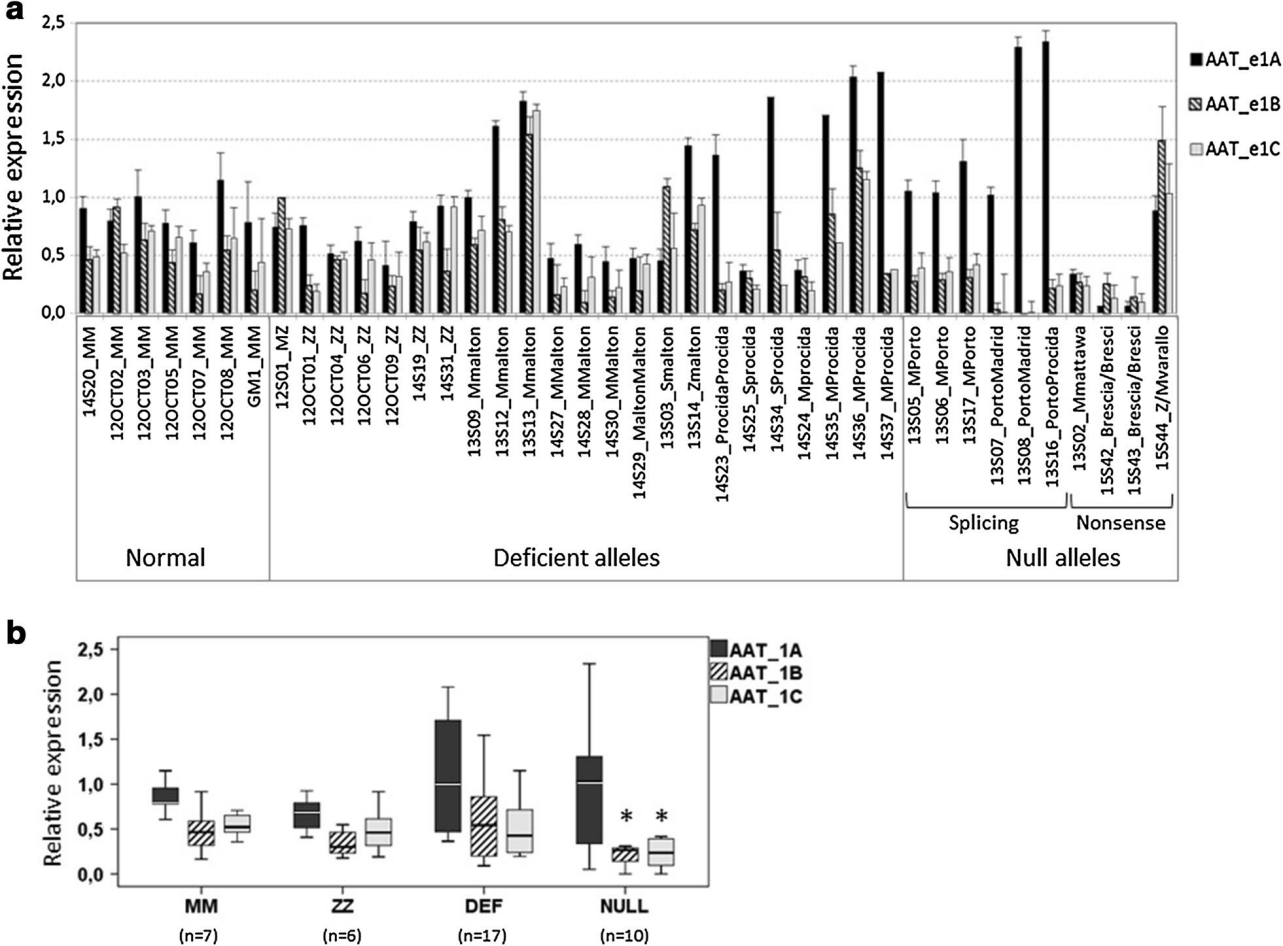
Transcriptional analyses in cases with normal, deficient or null alleles. **a** Relative expression of the AAT transcripts detected with the 1A, 1B or 1C assays in blood samples of subjects with AAT deficiency with deficient or null variants and normal samples (MM). **b** *Box plots* of the levels of expression of the 1A, 1B and 1C transcripts in groups of normal cases (MM), ZZ patients, cases with one or two deficient alleles other than Z (DEF), and cases with null alleles (NULL). Median expression is represented by the *horizontal line* within the *box*. Cases with null alleles showed statistically significant differences (*asterisk*) in the expression of 1B and 1C transcripts compared with expression in MM cases.

Three QT-PCR assays (1A, 1B and 1C, Figure 1) were applied on the peripheral blood samples from 33 AAT deficient subjects and 7 controls with normal MM genotype of AAT (Figure 4a). Subjects were carriers of heterozygous or homozygous variants of AAT (Z, S, MMalton, MProcida) or specific null alleles (QOMattawa, QOPorto, QOMadrid, QOBrescia or MVarallo) (Table 1). Notably, controls showed similar expression levels of transcripts detected either by 1A, 1B and 1C assays, although expression of the transcript I, detected by the 1A assay, was slightly higher as compared to 1B and 1C transcripts (Figure 4b). When compared to controls, ZZ patients showed no difference in the transcript levels. Interestingly, the level of 1A transcript appeared higher in cases carrying either one or two deficient AAT alleles (Figure 4b), and even higher in null cases caused by the splicing mutations but not by stop gain mutations (Figure 4a). Nevertheless, most cases with deficient alleles, such as heterozygous MMalton or MProcida alone or in combination with S or Z alleles, showed no significant changes in expression of the 1B and 1C transcripts (Figure 4b). This supports the notion that Z and other deficiency mutations do not affect the transcription of AAT. However, when compared to MM controls, null cases showed reduced levels of transcripts. Transcripts 1B and 1C were significantly reduced in carriers of either one or two null alleles with splicing mutations (QOPorto and QOMadrid) affecting the splicing donor site of intron 1C. In two cases carrying these two splicing mutations affecting both alleles, 1C transcripts were almost completely absent (Figure 4a). Null alleles caused by stop-gain mutations (QOMattawa and QOBrescia) showed substantial decrease in all transcripts. However, the null allele Mvarallo in combination with Z did not display any change in transcript levels.

### Correlation between expression of different transcripts and they association with AAT protein levels

The expression of transcripts detected with 1B and 1C assays showed a significant correlation (r = 0.83, p < 0.0001) in all analyzed cases. This finding suggests that (1) both assays are detecting the same transcription products and (2) the expression detected with the 1C assay comes from the monocyte transcription initiation sites but not from the hepatocyte specific transcript initiating in 1C exon. In contrast, the expression of 1B and 1C transcripts did not correlate with the different human tissues analyzed, indicating that the 1C expression, at least in lung, liver, kidney or pancreas tissues (Figure 2) comes from the hepatocyte transcription start site. The expression of 1A transcript did not correlate with 1B (r = 0.27, p = 0.104) or 1C (r = 0.25, p = 0.138) expression. Notably, in peripheral blood cells the 1A transcript was expressed at a higher level than 1B and 1C transcripts.

**Figure 5:**
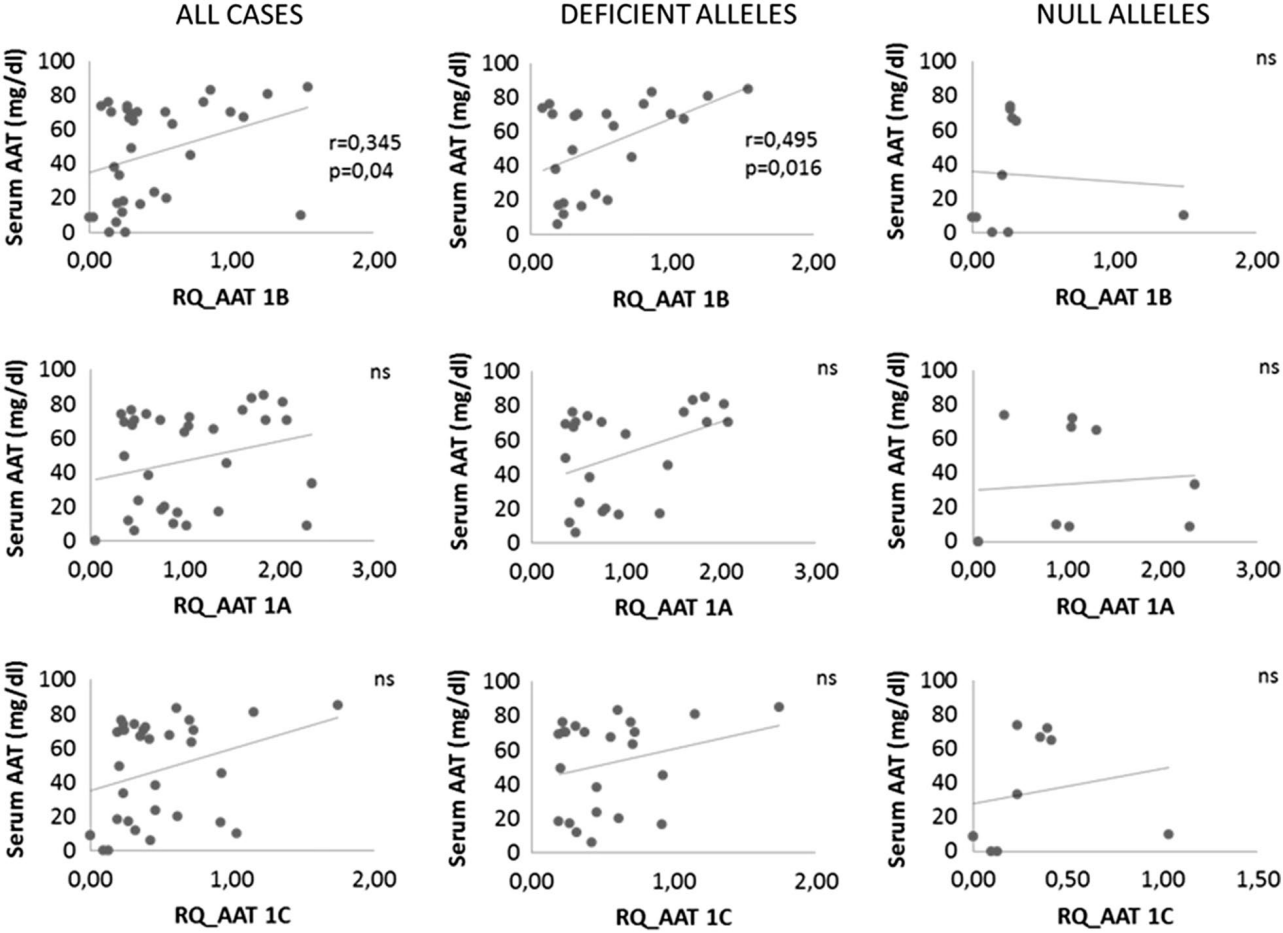
Correlation between expression level of transcripts and serum AAT levels in AAT deficient cases. *Panels* show correlation of expression of 1B transcripts (*upper row*), 1A (*middle row*) or 1C transcripts (*lower row*) with AAT levels in serum. Significant correlation was only found for 1B assay expression, both when all cases were analyzed together and in cases with deficient alleles, but not in cases with null alleles. *ns* Not statistically significant.

In all subjects we found no significant correlation between serum levels of AAT protein and mRNA of AAT by using 1A or 1C assays. In the other hand, AAT mRNA detected by the 1B assay showed a weak correlation with serum levels of AAT protein (r = 0.34, p = 0.04) (Figure 5). When 1B assay was applied for cases with deficient AAT alleles, levels of mRNA well correlated with serum AAT levels (r = 0.50, p = 0.016). Notably, this correlation did not exist in null cases.

## Discussion

Splicing variants have not yet been characterized for many genes and the contribution of most splicing events to human diseases, including AAT deficiency, remains unclear. The involvement of alternative splicing events in diagnosis and disease prognosis becoming increasingly recognized [17, 18]. This demands development of new methods to quantify the abundance of these isoforms in clinical samples. The QT-PCR is a “gold” standard method to analyze splicing variants; however it is limited by the difficulties in designing primers specific to splicing isoforms [30, 34, 35].

We have developed a new QT-PCR method, which allows analysis of the expression of different SERPINA1 transcripts generated by the alternative splicing between non coding exons 1A, 1B and 1C of the gene. Three QT-PCR assays were designed by using primers spanning the exon–exon junction (assay 1A) or primers flanking the alternatively spliced regions (assays 1B and 1C). Since some of the AAT noncoding exons are part of different transcript isoforms, separate quantification was only possible for transcripts with exon 1A joined to exon 2 (transcript I, Figure 1b). Nevertheless, we were able to estimate the abundance of transcripts containing different combinations of exons 1B or 1C.

The regulation of the expression of the SERPINA1 gene is complicated because of the existence of two different promoter regions with several transcription start sites and the phenomenon of alternative splicing, leading to the potential production of different SERPINA1 transcription products. At present 19 splice variants of the SERPINA1 are described in the Emsembl Genome Browser. Despite this large number of transcripts, only five of them represent main isoforms containing all coding exons and 3′UTR sequence with different combinations of non-coding 1A, 1B and 1C exons.

So far, the expression of SERPINA1 gene has been analyzed without taking into account different transcription products of the gene. Therefore, the role of the different mRNA SERPINA1 products in different cell types and tissues remains to be established. The analysis of different transcription products using our developed QT-PCR assays, permitted elucidation of the specific pattern of SERPINA1 transcripts in different tissues. The QT-PCR results revealed that transcripts containing exons 1B and 1C (transcripts III and IV, Figure 1b) are highly restricted to leukocytes. However, transcript containing exon 1A bond to exon 2 (transcript I), although expressed at high level in leukocytes, was also detected in lung tissue and less abundantly in liver, spleen, colon and prostate. Hence, the monocyte transcription start sites located within exon 1A seems to be active in other tissues. Nevertheless, currently we cannot dismiss the possibility that this expression comes from monocytes/macrophages present in the specific tissue.

Expression of different transcripts in lung, liver, kidney, pancreas, spleen, colon, small intestine and prostate detected by the 1C assay was higher than detected by 1A and 1B assays. We believe that the 1C expression in these tissues reflects the expression of transcript V initiated in the hepatocyte transcription start site. Indeed, transcripts from 1C might be specifically expressed in extra-hepatic tissues. In fact, this was previously reported in transgenic mice studies [36–38]. Further studies in isolated cell types are warranted to elucidate the biological role of these different transcripts.

Furthermore, we found changes in the expression of specific transcripts in blood samples from subjects with AAT deficiency variants. As a matter of fact, null alleles produced by premature stop codons showed reduced levels of all transcripts. This latter could be explained by the activation of the nonsense mediated decay (NMD) mechanism typically occurring in null cases [39, 40]. This was specifically found in homozygous QOBrescia/QOBrescia cases and even in the M/QOMattawa. In null alleles caused by splicing mutations (QOPorto and QOMadrid) the reduction in 1B and 1C transcript levels might be explained by the fact that these splicing variants are localized in the donor splice site of exon 1C. It is probable that other null alleles, typically produced by premature stop codons, may have reduced levels of transcription. Supporting this assumption some researchers have found important effects of different AAT null mutations on the AAT mRNA level, such as QOGranite falls or QOTrastevere [41]. In general, cellular content of AAT mRNA is assumed to be affected by the distance of the nonsense codon from the initiator codon, although elements within the coding region may also have importance in conferring mRNA stability. Because transcription depends on the mutation, it is important to investigate whether other AAT variants (deficient and null) affect 1A, 1B or 1C transcription levels. Our QT-PCR approach might help to identify how mutations affect transcription of the AAT gene.

Remarkably, subjects with deficient AAT alleles like Z, S, MMalton, and MProcida showed no significant differences in 1A, 1B or 1C transcription levels relative to normal MM cases. Pathogenesis of the deficient variants of AAT is typically characterized by intracellular accumulation of AAT and a concomitant reduction in its circulating levels but not by the reduction in protein synthesis. Therefore, one cannot expect significant changes to occur at the mRNA level of the transcripts.

As AAT is an acute phase reactant, its expression increases during inflammatory responses. Although AAT is primarily synthesized in the liver [4], other cells also can produce lower amounts of AAT. Basal and modulated expression of AAT is controlled by many different cis and trans-acting factors that can substantially activate transcription [7, 11, 42, 43]. The increased secretion of AAT is mainly mediated by the cytokines, such as IL-6, IL-1β, TNFα, oncostatin M or bacterial lipopolysaccharide [21, 42, 43]. These stimuli seem to act specifically on hepatocytes, monocytes, macrophages or other AAT producing cells, and to induce expression of AAT by the different magnitude [29]. How all these factors affect expression, stability or translation rates of the different SERPINA1 transcripts remains to be elucidated.

## Conclusion

We developed three QT-PCR assays to quantify the expression of SERPINA1 transcripts. By using these assays we quantified specific expression of transcripts in various tissues and found that transcript expression is affected by the deficient and null variants of SERPINA1 gene. The possibility to distinguish specific alternative transcripts opens new possibilities to explore the clinical importance of SERPINA1 gene mutations. Moreover, it helps to build a better understanding of regulation mechanisms of SERPINA1 gene expression. To understand the function of individual transcript variants in isolated cells from individuals with AAT deficiency will be the next step of our investigations.

## Additional file

**Additional file 1.** Estructure of the main transcripts of SERPINA1 gene and their corresponding transcripts in Ensemble.

## Abbreviations

AAT: alpha-1 antitrypsin
QT-PCR: quantitative polymerase chain reaction
RT-PCR: reverse transcription polymerase chain reaction amplification
5′UTR: 5′ untranslated region
NMD: nonsense mediated decay
