# Supplementary material for: Alternative transcripts of the SERPINA1 gene in alpha-1 antitrypsin deficiency
Source: J Transl Med. 2015 Jul 4;13:211. doi: 10.1186/s12967-015-0585-y (PMC4490674; doi:10.1186/s12967-015-0585-y)
Supplement: Supplementary file 1 — Additional file 1. Estructure of the main transcripts of SERPINA1 gene and their corresponding transcripts in Ensemble. [file 12967_2015_585_MOESM1_ESM.docx]

| **1A-e2 (assay 1A)** |  |  |  | |  |  |
| --- | --- | --- | --- | --- | --- | --- |
| **Assay details:** | | | | | | |
|  | | | | | | |
| **Use Universal ProbeLibrary probe: #18** | | | | | | |
| **Primer** | **Length** | **Position** | | **Tm** | **%GC** | **Sequence** |
| Left Primer | 20 | 165 - 184 | | 59 | 55 | tgaggagagcaggaaaggac |
| Right Primer | 18 | 243 - 260 | | 59 | 67 | ctcagccagggagacagg |
| **Amplicon (96 nt)** | | | | | | |
| tgaggagagcaggaaag/gacaatgccgtcttctgtctcgtggggcatcctcctgctggcaggcctgtgctgcctggtccctgtctccctggctgag | | | | | | |
|  |  |  |  | |  |  |
| **1C-e2 (assay 1C)** |  |  |  | |  |  |
| **Assay details:** |  |  |  | |  |  |
| **Use Universal ProbeLibrary probe: #73** | | | | | | |
| **Primer** | **Length** | **Position** | | **Tm** | **%GC** | **Sequence** |
| Left Primer | 21 | 191 - 211 | | 59 | 48 | ttaaatacggacgaggacagg |
| Right Primer | 20 | 265 - 284 | | 59 | 50 | acgagacagaagacggcatt |
| **Amplicon (94 nt)** | | | | | | |
| ttaaatacggacgaggacagggccctgtctcctcagcttcaggcaccaccactgacctgggacagtgaatc/gacaatgccgtcttctgtctcgt | | | | | | |
|  |  |  |  | |  |  |
|  |  |  |  | |  |  |
| **1B-1C (assay 1B)** |  |  |  | |  |  |
| **Assay details:** |  |  |  | |  |  |
| **Use Universal ProbeLibrary probe: #39** | | | | | | |
| **Primer** | **Length** | **Position** | | **Tm** | **%GC** | **Sequence** |
| Left Primer | 23 | 381 - 403 | | 59 | 52 | cagctaagtggtactctcccaga |
| Right Primer | 21 | 492 - 512 | | 60 | 48 | tcgtccgtatttaagcagtgg |
| **Amplicon (132 nt)** | | | | | | |
| cagctaagtggtactctcccagagactgtctgactcacgccaccccctccaccttggacacaggacgctgtggtttctgagccag/cagcctcccccgttgcccctctggatccactgcttaaatacggacga | | | | | | |
|  |  |  |  | |  |  |

**Title: Alternative transcripts of the SERPINA1 gene in Alpha-1 Antitrypsin Deficiency**

**Supplementary material.**

Description of the three QT-PCR assays, including the sequences of the amplicons used to identify the regions 1A-E2 (assay 1A), the 1C-E2 (assay 1C) and the region 1B-1C (assay 1B). Probes 18, 73 and 39 (in red) were used for assays 1A, 1C and 1B respectively. Probe 18 is localized in exon 2, Probe 73 is localized in exon 1C, and Probe 39 localized in exon 1B. Separations between exons are marked with a slash (/).
